# Absence of infratentorial lesions in Fabry disease contributes to differential diagnosis with multiple sclerosis

**DOI:** 10.1002/brb3.1121

**Published:** 2018-10-02

**Authors:** Lorenzo Ugga, Sirio Cocozza, Giuseppe Pontillo, Camilla Russo, Vincenzo Brescia Morra, Roberta Lanzillo, Eleonora Riccio, Antonio Pisani, Arturo Brunetti

**Affiliations:** ^1^ Department of Advanced Biomedical Sciences University of Naples “Federico II” Naples Italy; ^2^ Department of Neurosciences and Reproductive and Odontostomatological Sciences University of Naples “Federico II” Naples Italy; ^3^ Department of Public Health, Nephrology Unit University of Naples “Federico II” Naples Italy

**Keywords:** Fabry disease, infratentorial, MRI, multiple sclerosis

## Abstract

**Background and Purpose:**

Multiple Sclerosis (MS) has been proposed as a possible differential diagnosis with Fabry Disease (FD). We evaluated the incidence of infratentorial lesions in FD patients, investigating whether their presence could help in differentiating these two conditions. We explored the diagnostic accuracy of this sign alone and in combination to the involvement of corpus callosum (CC).

**Methods:**

White Matter lesions were retrospectively evaluated on FLAIR images available from 136 MS and 144 FD patients. Infratentorial involvement was assessed considering the whole cerebellum, and the part of the brainstem included between the occipital foramen and the upper edge of the red nucleus. Furthermore, the presence of callosal lesions was also recorded, evaluating the portion of CC included between the two external walls of the lateral ventricles.

**Results:**

Infratentorial involvement was detectable in 119/136 (87.5%) MS patients, while it was present in only 17/144 (11.8%) FD patients. When the diagnostic performance of a positive infratentorial involvement was evaluated in combination with the presence of CC lesions, a specificity of 97%, with a positive predictive value of 96% was reached.

**Conclusions:**

We concluded that the absence of infratentorial lesions, especially when combined to the evaluation of other typical imaging features, can help in the differential diagnosis between MS and FD.

## 1 INTRODUCTION

Fabry Disease (FD) is a rare X‐linked lysosomal storage disorder caused by abnormalities in the α‐galactosidase A gene which leads to a lack or deficiency of this enzyme, with progressive accumulation of glycosphingolipids in different tissues (Zarate & Hopkin, 2008).

Central Nervous System (CNS) involvement is common, and it is usually characterized on MRI by the presence of single, multiple, or confluent white matter lesions (WMLs) on T2‐weighted images (Fellgiebel, Müller, & Ginsberg, 2006). This feature, coupled to the high heterogeneity of clinical presentations, conducted to the suggestion that FD could be considered in specific cases as a possible differential diagnosis of Multiple Sclerosis (MS) (Böttcher et al., 2013; Callegaro, Lino, & Kaimen‐Maciel, 2006).

In this setting, the search for radiological findings that can allow a differential diagnosis between these two conditions is essential, since a wrong diagnosis could influence treatment and patients’ management.

Recently, it has been reported that the involvement of corpus callosum (CC) in FD patients is lower than those detectable in subjects affected by MS, independently from the clinical presentation and the WMLs load, suggesting its evaluation as an accessory radiological finding that could help in differential diagnosis between the two conditions (Cocozza, Olivo, et al., 2017).

Infratentorial involvement is common in MS patients, being also an important predictor of long‐term disability (Mormina et al., 2017) and allowing to demonstrate dissemination in space, according to the diagnostic criteria (Polman et al., 2011).

To date, no information about the incidence of infratentorial lesions in FD has been reported.

The aim of our study was to (a) evaluate the incidence of infratentorial lesions in FD patients and (b) investigate whether their evaluation could be an additional tool to differentiate FD from MS, exploring the diagnostic accuracy of this sign alone and in combination to the callosal involvement.

## 2 METHODS

In this retrospective study, we evaluated MRI scans from 144 FD and 136 MS patients. All FD patients received a genetically confirmed diagnosis, while all MS encompassed all clinical phenotypes and fulfilled the 2010 revised McDonald criteria (Polman et al., 2011).

For all subjects included in the analysis, two neuroradiologists blinded for the diagnosis rated in consensus the presence and the load of WMLs, defined as areas of increased signal on T2‐weighted images within brain parenchyma, in periventricular and deep hemispheric white matter on FLAIR images, acquired on different scanners and strength fields. In particular, 28 out of 144 FD patients (19.4%) were acquired on 0.5 T scanners, 70/144 (48.6%) subjects were scanned on MR systems at 1.5 T, with the remaining 46 patients (32.0%) acquired on 3 T MR machines. On the other hand, 85 MS patients (62.5%) were acquired on 3 T scanners, with the remaining 51 subjects (37.5%) that underwent MR examination at 1.5 T, since no patient was examined using 0.5 T scanners.

In all patients, the evaluation of WMLs was performed on both fluid‐attenuated inversion recovery (FLAIR) sequences and T2‐weighted sequences, all acquired with a slice thickness ≤5 mm. For all subjects, image assessment was achieved independently from the acquisition orientation with at least two planes that were evaluated, without following any specific order in the evaluation.

According to previous reports (Cocozza, Olivo, et al., 2017; Cocozza, Russo, et al., 2017), a modified Fazekas score (Fazekas et al., 2015) was used to identify the presence of WMLs, evaluating two different locations (periventricular and deep hemispheric white matter), each ranging from 0 (indicating absence of WMLs) to 3 (suggesting high WMLs load), with a total score that ranged from 0 to 6.

The incidences of CC and infratentorial involvement were then probed for each group of subjects.

Callosal involvement assessment was performed similarly to what previously described (Cocozza, Olivo, et al., 2017), with the CC that was evaluated considering commissural fibers from the midline to a vertical plane passing through the external wall of the lateral ventricle (at the level of the cella media) on both sides, while the infratentorial involvement was assessed considering the whole cerebellum and the part of the brainstem included between the occipital foramen and the upper edge of the red nucleus.

### 2.1 Statistical analysis

All statistical analyses were performed using Statistical Package for Social Science package (SPSS Inc.,v.17.0,Chicago, IL), and the level of significance was set at *p* < 0.05 for all tests. An unpaired *t* test was used for comparing ages and Fazekas scores, while a chi‐squared test was used to determine differences in terms of sex. Measures of diagnostic performance were calculated for the different proposed imaging findings. In particular, odds ratios were determined as an overall indicator of test performance, with differences between the single diagnostic criteria that were probed using McNemar’s test.

## 3 RESULTS

The FD and MS groups were not significantly different in terms of sex, (M/F = 57/87 vs. M/F = 42/94; FD vs. MS, *p* = 0.128), while the FD group proved to have a mean age higher than those found in the MS group (42.1 ± 14.0 vs. 38.1 ± 10.9, for FD and MS, respectively; *p* = 0.008). A complete depiction of the patients’ demographic and clinical characteristics is provided in Table 1.

**Table 1:** Subject demographics and clinical variable of all patients included in the study

	MS (*n* = 136)	FD (*n* = 144)
Age (mean ± *SD*)	38 ± 10.9 (range 12–65)	42 ± 14.0 (range 13–73)
Sex (M/F)	42/94	57/87
Neurological involvement	–	70/144
Cardiac involvement	–	67/144
Renal failure	–	53/144
Proteinuria	–	61/144
ERT	–	90/144
DD (mean ± *SD*)	10.4 ± 7.4	–
EDSS (median)	3.0 (range 1.5–7.0)	–

Notes

DD: disease duration; EDSS: expanded disability status scale; ERT: enzyme replacement therapy; FD: Fabry disease; MS: multiple sclerosis; *SD*: standard deviation.

Ages and DD are expressed in years.

In FD patients, neurological involvement was defined as positive if central or peripheral nervous system symptoms were present (including stroke, acroparesthesia, cephalalgia, etc.). Similarly, cardiac involvement was considered positive if arrhythmia or left ventricular hypertrophy were present.

Renal failure was considered present with an estimated glomerular filtration rate <90 ml/min, while proteinuria was considered positive for values >150 mg/24 hr.

Among the total of 144 FD patients, WMLs were detected in 71 subjects (49.3%), with a mean Fazekas score of 2.2 (±1.56), while all MS patients showed the presence of WMLs, with a mean Fazekas score of 2.6 (±1.26).

For the MS group, CC lesions were present in 121/136 subjects (89.0%), with infratentorial involvement detectable in 119/136 (87.5%) patients. On the other hand, in FD patients, only eight subjects on 144 (5.6%) proved to have a CC lesion, with 17/144 (11.8%) that showed an infratentorial involvement.

The involvement of at least one of these two locations was proved in 128/136 MS patients (94.1%) and 21 out of 144 (14.6%) subjects with FD. On the other hand, the absence of callosal and infratentorial involvement was found only in 8/136 subjects affected by MS (5.9%), while in the 85.4% of cases (123/144 subjects), FD patients were spared by WMLs in these regions. Finally, when both signs were considered, MS patients proved to be positive in the 82.4% of the cases (112/136 patients), while only four FD patients (2.8%) showed the contemporary presence WMLs in CC and the infratentorial area (Table 2).

**Table 2:** Incidence of the evaluated MRI signs in Multiple Sclerosis and Fabry disease patients

	MS (*n* = 136, %)	FD (*n* = 144, %)
Corpus callosum involvement	121 (89.0)	8 (5.6)
Infratentorial involvement	119 (87.5)	17 (11.8)
Corpus callosum + Infratentorial involvement	112 (82.4)	4 (2.8)
Involvement in at least one area	128 (94.1)	21 (14.6)
No involvement	8 (5.9)	123 (85.4)

Note

FD: Fabry disease; MS: multiple sclerosis.

In line with this last finding, the evaluation of diagnostic performances showed that the combination of both CC and infratentorial involvement reached a specificity of the 97%, with a positive predictive value of 96% and a positive likelihood ratio of 27.3. Similarly, the evaluation of at least one location proved to be useful to exclude MS from the possible differential diagnosis with FD, scoring a sensitivity, negative predictive value, and negative likelihood ratio of 94%, 93%, and 0.07, respectively (Table 3).

**Table 3:** Measures of diagnostic performance for the evaluated MRI signs in Multiple Sclerosis and Fabry disease patients

	Corpus callosum involvement	Infratentorial involvement	Corpus callosum + Infratentorial involvement	Involvement in at least one area
Sensitivity (%)	89	87	82	94
Specificity (%)	94	88	97	85
Positive predictive value (%)	94	87	96	86
Negative predictive value (%)	90	88	85	93
Accuracy (%)	92	88	90	90
Positive likelihood ratio	14.8	7.2	27.3	6.3
Negative likelihood ratio	0.12	0.15	0.18	0.07
Diagnostic odds ratio	137.1	52.3	163.3	93.7

The OR analysis confirmed that the best overall diagnostic performance was achieved when considering both CC and infratentorial involvement. Indeed, the simultaneous presence of both CC and infratentorial lesions corresponded to an OR of 163.3 was significantly higher compared to the sole evaluation of the involvement of the CC (OR = 137.1; *p* < 0.001) or of the infratentorial region (OR = 52.3; *p* < 0.001), as well as the positivity of least one of the two areas (OR = 93.7; *p* < 0.001; Table 3).

## 4 DISCUSSION

Due to the heterogeneity of clinical and radiological presentations in MS, FD has been recently proposed as a possible under‐recognized diagnosis in some MS cases (Böttcher et al., 2013). Although a careful neurological examination, along with an accurate evaluation of systemic involvement, is undoubtedly central to achieve a correct diagnosis (Colomba et al., 2018), neuroimaging plays an essential role in the diagnostic workup of these patients.

In this setting, our results expand the current knowledge about the differential diagnosis between MS and FD, suggesting that infratentorial lesions are much less common in FD compared to MS. However, their role as radiological tool for the differential diagnosis between these two conditions is less significant compared to the evaluation of CC, which apparently remains the best indicator for differential diagnosis between FD and MS. Nevertheless, it should be noted that when both these two signs were found positive, high positive predictive value (96%) and specificity (97%) were achieved, with a minor loss in negative predictive value (85%) and sensitivity (82%) compared to those achievable when the two regions were evaluated alone.

**Figure 1:**
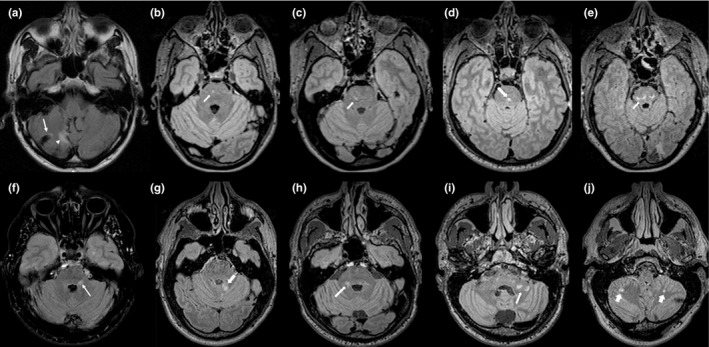
Axial FLAIR images showing the different patterns of infratentorial lesions in five patients with Fabry disease (a–e) and five subjects affected by Multiple Sclerosis (f–j). In FD, a typical vascular appearance is present (a), with the preferential involvement of the central portions of the pons (b‐e). On the other hand, in MS infratentorial lesions resemble classic demyelinating plaques, with the characteristic involvement of the middle cerebellar peduncles (f–i) or the cerebellar white matter (j)

**Figure 2:**
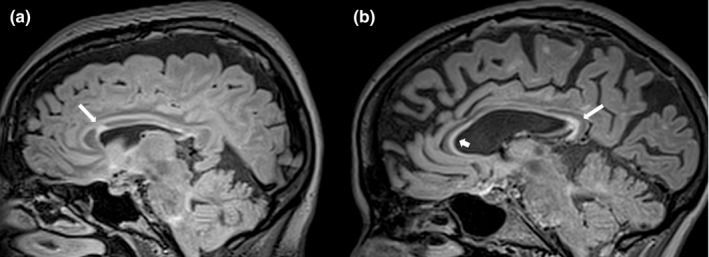
Parasagittal FLAIR images showing the appearance of Corpus Callosum lesions in a patient with Fabry Disease (a) and in one with Multiple Sclerosis (b). In the MS patient is possible to better appreciate, compared to FD, the typical appearance of the calloso‐septal lesions, which are defined as narrow hyperintense bands along the undersurface of the corpus callosum itself

It should be noted that in those cases where FD patients showed an infratentorial or callosal involvement on T2‐weighted image, lesions did not resemble the typical appearance of a demyelinating plaque, both in terms of anatomical distribution and morphological appearance (Figures 1 and 2). This observation suggests that the presence/absence of an infratentorial or callosal lesions is not the only variable that should be taken into account in doubt cases. Indeed, lesion morphology and location could further address the neuroradiologist toward a correct diagnosis, reflecting the different etiology of the lesion (vascular vs. demyelinating). In this context, the evaluation of other typical MS lesions sites, such as juxtacortical or cortical lesions, although providing a specific sign of pathology is less practical, reproducible, and easy‐to‐identify neuroradiological signs compared to those here proposed. Similarly, the central vein sign has been proposed as a novel MRI biomarker that could improve the accuracy of MS diagnosis (Campion et al., 2017; Maggi et al., 2018; Sati et al., 2016). This sign, defined as the presence of an area of hypointensity, corresponding to a venule, in the context of a T2‐weighed hyperintense lesion, is displayable on FLAIR* sequences (obtained combining T2*‐weighted and FLAIR images). It is reasonable to hypothesize that the evaluation of the central vein sign could provide an additional MRI feature to differentiate between FD and MS, showing a different behavior between these two conditions, which could reflect the different etiology. However, due to the retrospective nature of this study, we were not able to obtain this information from our dataset, leaving this hypothesis still uninvestigated. Future prospective studies are strongly warranted, to investigate the possible role of the central vein sign in differentiating between FD and MS.

It is also reasonable to hypothesize how a different behavior in terms of gadolinium enhancement between these two conditions, again reflecting the different etiology behind WMLs development in FD and MS, could help in their differential diagnosis. In particular, the presence of enhancing lesions is expected in MS patients, being also a way to demonstrate dissemination in time according to the diagnostic criteria (Polman et al., 2011). On the other hand, blood‐brain barrier is supposed to remain almost intact in FD patients (Böttcher et al., 2013), therefore offering a possible imaging feature able to discriminate between these two conditions. Nevertheless, a significant renal impairment is present in FD patients (Pisani et al., 2014). This clinical finding, coupled to the recent concerns raised about a possible gadolinium deposition in the brain of patients undergoing multiple contrast MRI exams (Tedeschi et al., 2017), indirectly support the use of feasible and easy‐to‐identify neuroradiological signs, such as the combined callosal‐infratentorial evaluation, to differentiate FD from MS.

Finally, it is noteworthy to mention the possible use of advanced image analysis techniques, such as the creation of lesion probability maps, to investigate the possible different spatial distribution of WMLs in FD and MS. Indeed, it can be hypothesized a possible further increase in the ability to discriminate between these two conditions using this approach, with a different anatomical lesion localization reflecting the different pathophysiology of the damage. However, this speculation remains to be tested, and future studies comparing lesion probability maps between FD and MS patients are warranted.

Our results were obtained in a large and representative population of FD and MS patients. In particular, the last‐mentioned group was composed by patients encompassing all clinical phenotypes. It could be therefore hypothesized a possible increase in the detection of WML in this group due to the presence of patients with a long disease duration (DD) and, accordingly, a higher WML load. This is remarkable, since it is reasonable to hypothesize that a differential diagnosis between FD and MS could arise especially in the earliest phases of the MS, where clinical and radiological findings can range from mild to moderate. However, an ancillary analysis carried out on a subgroup of MS patients with <3 years of DD showed a similar incidence of callosal and infratentorial involvement between early patients and the entire MS group (data not shown), indirectly strengthening the role of these neuroradiological signs.

Some limitations should be considered in the present report. In particular, due to the retrospective nature of the study, MR scans were performed at different field strengths, thus possibly limiting the identification of lesions in some subjects. In particular, a subgroup of FD patients (<20%) underwent MR examination on 0.5 T systems, while no MS patients were studied using scanners with similar field strength. Similarly, a higher proportion of MS patients underwent an MRI exam on high‐field scanner compared to the FD population (62.5% vs. 32.0%), thus possibly overestimating and underestimating WMLs in the MS and FD groups, respectively. For this reason, future studies are needed to collect a higher number of patients acquired with similar MRI sequences, possibly on scanners of the same field intensity.

## 5 CONCLUSIONS

In conclusion, FD patients show a relatively low incidence of infratentorial lesions. Although configuring a less powerful diagnostic tool than sole evaluation of CC involvement, its combination with the latter can increase some diagnostic measures for differential diagnosis between MS and FD, further allowing the physician to avoid misdiagnosis, providing subsequent correct and prompt treatment options.

## INFORMED CONSENT

Informed consent was previously obtained from all individual participants included in the study.

## ETHICAL APPROVAL

For this type of retrospective study, formal consent is not required.

## CONFLICT OF INTEREST

SC and CR received fees for speaking by Genzyme. AP received reimbursement for attending symposiums, fees for speaking, funds for research, and fees for consulting by Shire, Genzyme, and Amicus. All other authors declare no conflict of interest.
